# Supplementary material for: Seminoma and Embryonal Carcinoma Footprints Identified by Analysis of Integrated Genome-Wide Epigenetic and Expression Profiles of Germ Cell Cancer Cell Lines
Source: PLoS One. 2014 Jun 2;9(6):e98330. doi: 10.1371/journal.pone.0098330 (PMC4041891; doi:10.1371/journal.pone.0098330)
Supplement: File S1 — ZIP file containing DMRforPairs output for significant regions. Please start from the html files. (ZIP) [file pone.0098330.s008.zip › figures/35577.pdf]

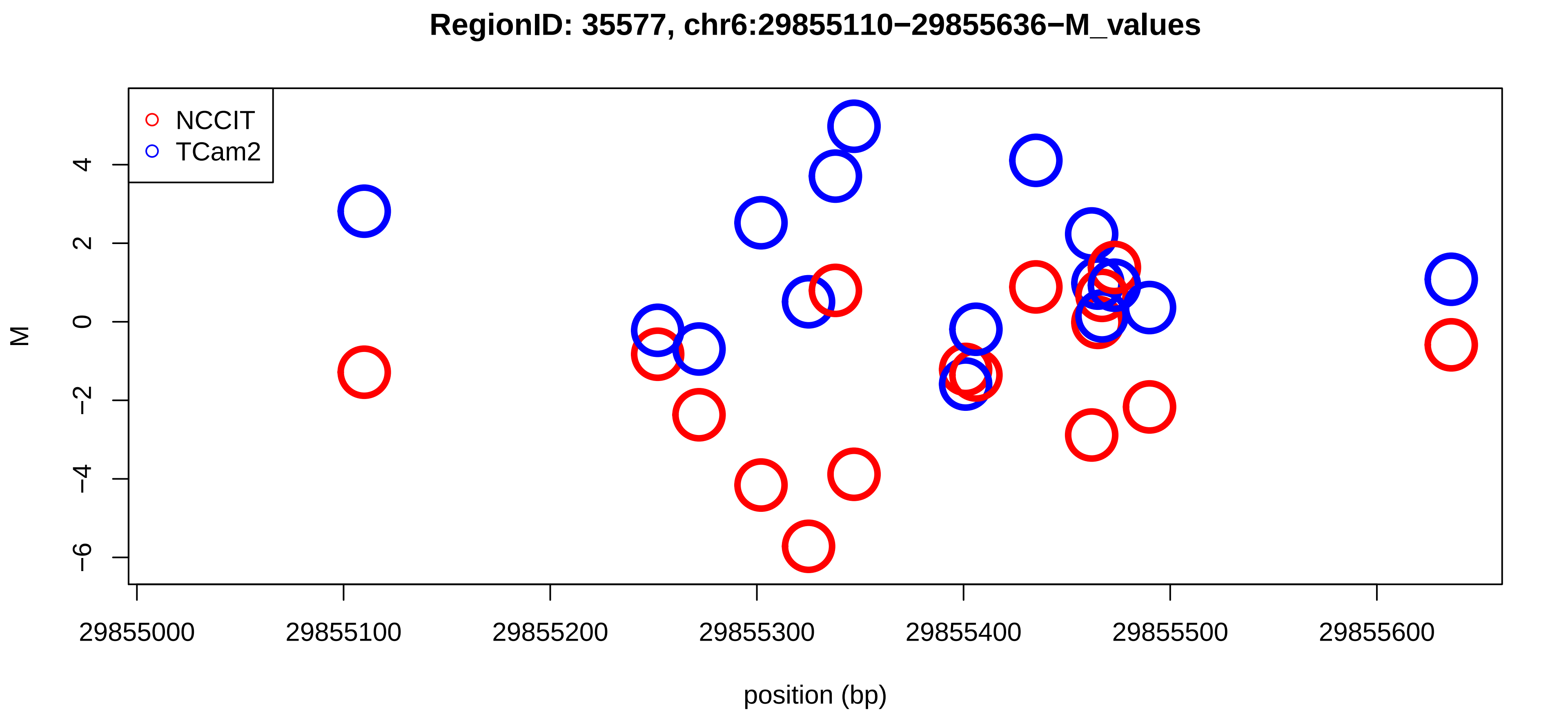
RegionID: 35577, chr6:29855110−29855636−M_values
NCCIT
TCam2
M
position (bp)
29855000
29855100
29855200
29855300
29855400
29855500
29855600
−6
−4
−2
0
2
4

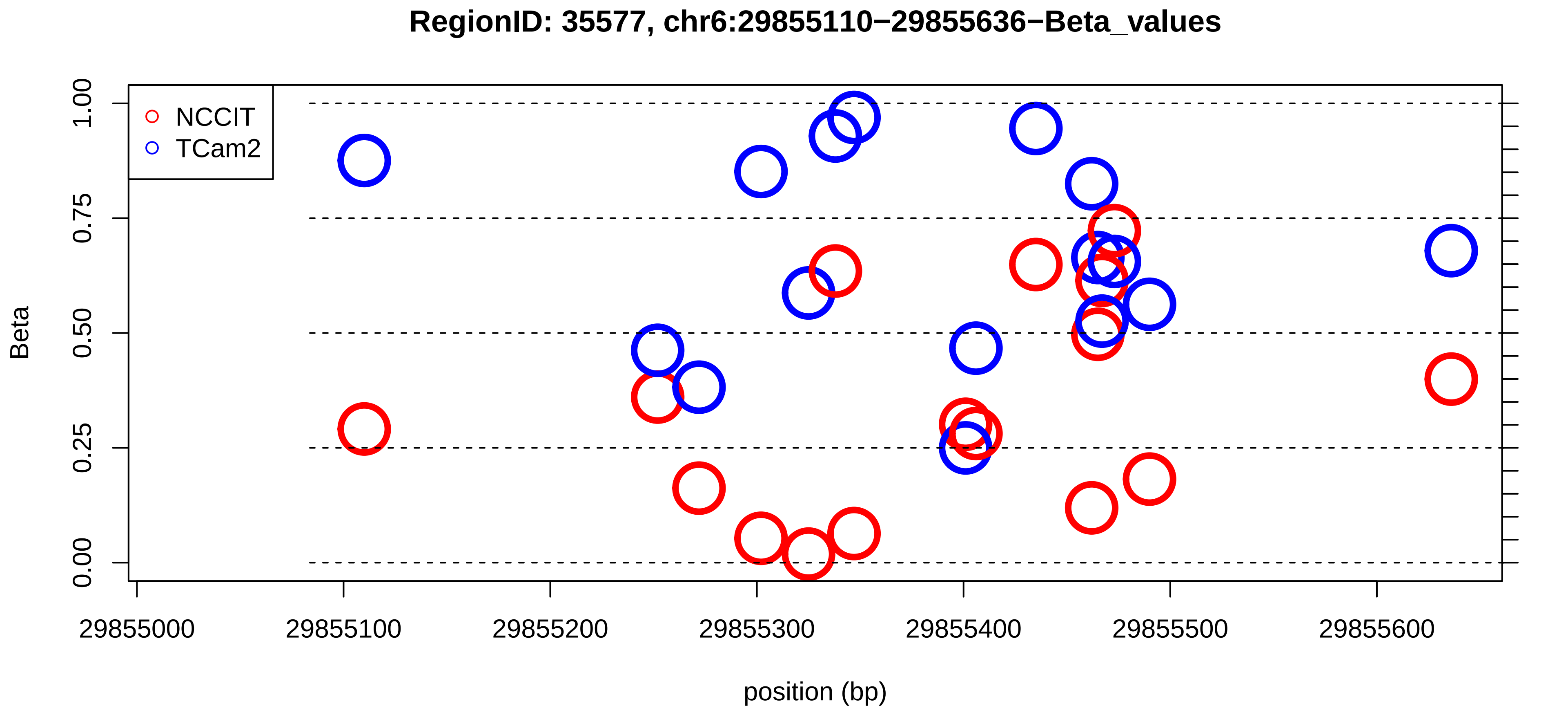
RegionID: 35577, chr6:29855110−29855636−Beta_values
NCCIT
TCam2
Beta
position (bp)
29855000
29855100
29855200
29855300
29855400
29855500
29855600
0.00
0.25
0.50
0.75
1.00
